# Imatinib-induced hyperpigmentation

**DOI:** 10.1016/j.jdcr.2024.06.038

**Published:** 2024-07-21

**Authors:** Caroline Garraway, Kevin Yang, Kavita Kantamneni, Rebecca Kissel, Peter G. Pavlidakey

**Affiliations:** a Department of Dermatology, University of Alabama at Birmingham, Birmingham, Alabama; b UAB Heersink School of Medicine, Birmingham, Alabama

**Keywords:** drug-induced pigmentation, hyperpigmentation, imatinib

## Introduction

Imatinib is a tyrosine kinase inhibitor used in several malignancies, including chronic myeloid leukemia. Imatinib acts on multiple targets, including BCR-ABL, c-KIT, platelet-derived growth factor receptor, and colony-stimulating factor receptor-1.^1^ Multiple cutaneous adverse effects have been reported with administration including nonspecific rash, pruritus, hypopigmentation, and rarely, hyperpigmentation, particularly of the oral mucosa.^1^ We present a case of significant blue-gray hyperpigmentation of the face and trunk.

## Case report

**Fig 1:**
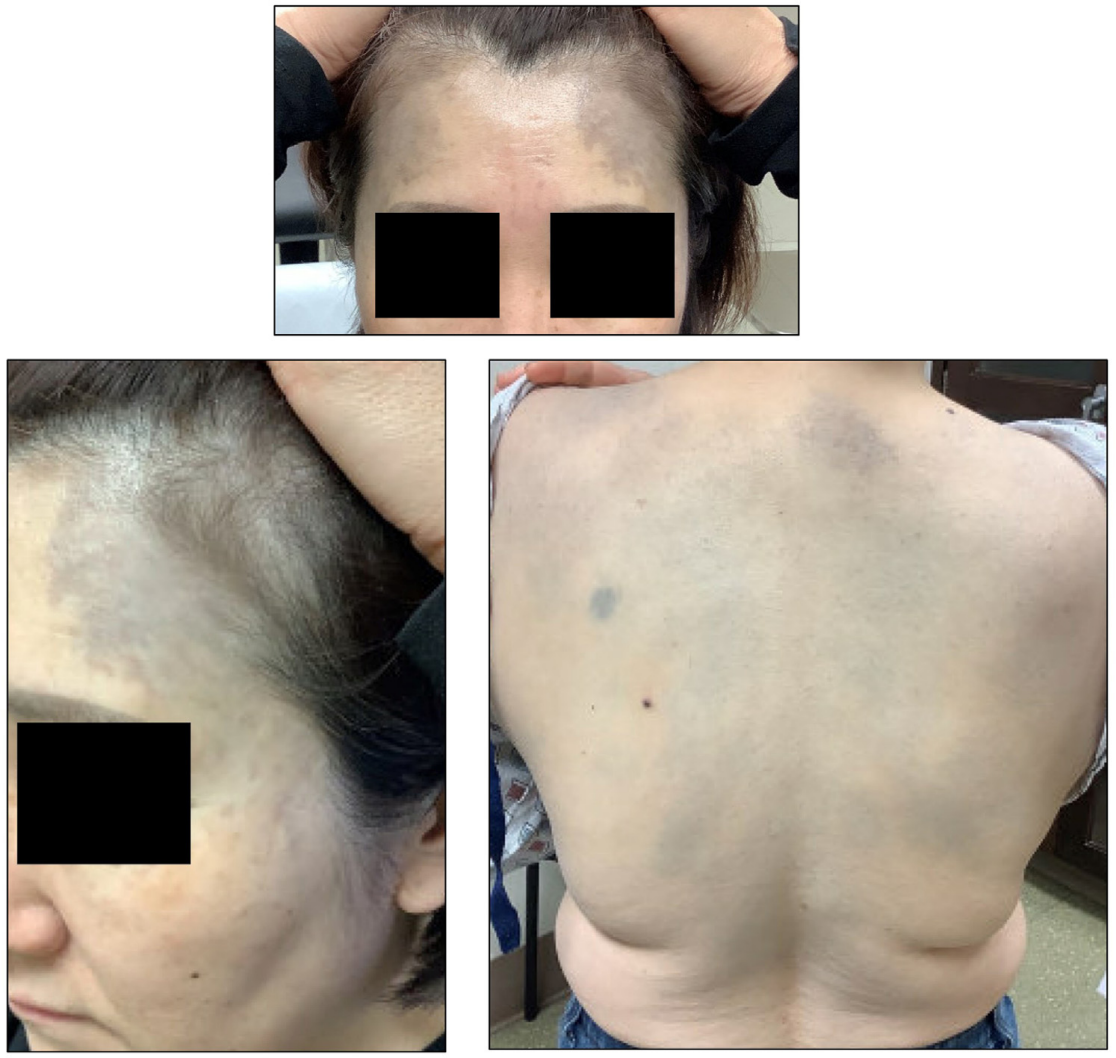
Mottled blue-gray pigmented patches of the bilateral temples and back.

A 46-year-old Korean woman with a history of chronic myeloid leukemia presented to dermatology clinic with progressive asymptomatic discoloration of the forehead, back, and right arm of 3-year duration. She began taking imatinib approximately 16 years prior to presentation for chronic myeloid leukemia and denied taking any other medications. Physical examination showed mottled blue-gray hyperpigmented patches of the bilateral temples extending posteriorly to the frontal scalp. Similar ill-defined blue-gray patches were scattered across diffusely on the back with accentuation around the middle portion (Fig 1). There was no mucosal or nail involvement noted on examination.

**Fig 2:**
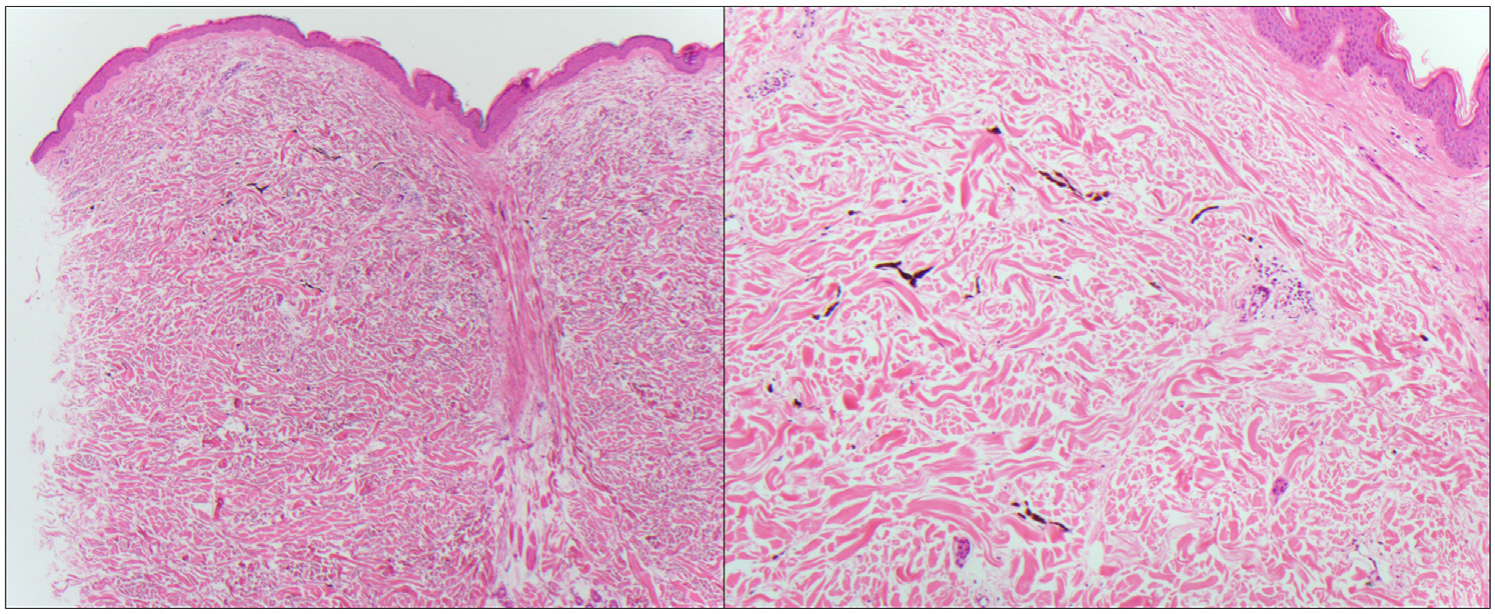
Hematoxylin-eosin (H&E) staining demonstrated dark-brown pigment in melanophages perivascularly and around eccrine coils in the dermis.

A biopsy was performed of the patient’s back with histopathology demonstrating deposition of dark-brown pigment in melanophages and dendritic mononuclear cells throughout the dermis in a perivascular and peri-eccrine distribution (Fig 2). Additional staining was negative for iron and positive for melanin highlighted by Fontana Masson. Immunostaining with SOX10 demonstrated retained melanocytes within the epidermis with a lack of dermal staining. Overall, pathology results were suggestive of medication-induced pigment deposition secondary to imatinib. Imatinib was subsequently discontinued by the oncology team but patient declined further management of the hyperpigmentation.

## Discussion

Hypopigmentation is known to be a side effect of imatinib; this is because of the role of c-KIT in proliferation and survival of melanocytes.^2^ Conversely, hyperpigmentation is a rare side effect of imatinib therapy and in most cases has been reported in involvement of oral mucosa, teeth, and nails.^3^ Cases affecting the skin have rarely been reported in the literature with varying degrees of pigmentation ranging from small hyperpigmented macules to localized patches distributed on the face or back.^4^, ^5^, ^6^ In 1 case, a patient with vitiligo had repigmentation of depigmented lesions after initiation of imatinib therapy for gastrointestinal stromal tumors.^7^ In our case, the body surface area involvement was greater than previously reported cases, likely because of the long duration over which the patient had been taking imatinib as well as the 3-year delay in presentation since the initial onset of the pigmentation.

The mechanism for hyperpigmentation is unclear, and multiple histologic patterns of pigment deposition have been identified, reflecting various possible mechanisms. Alexandrescu et al^5^ demonstrated a case with increased basal layer pigmentation. Kagimoto et al^6^ reported a case of hyperpigmentation with pathology showing pigment incontinence secondary to a lichenoid drug eruption. In fact, other tyrosine kinase inhibitors such as nilotinib and dasatinib have been associated with eruption of follicular lichen planus and lichen planopilaris lesions, suggesting the consideration of lichen planus pigmentosus given the distribution of the pigmentation and some of the histologic findings.^8^ In contrast, there was lack of inflammatory infiltrate noted on histology despite clinical progression of pigmentation. Furthermore, the depth of pigmentation in the dermis of our case was greater than would be expected for postinflammatory pigment alteration. In cases from Singapore and South Korea, histologic patterns showed dermal melanocytes, consistent with acquired dermal melanocytosis.^9^ Others have suggested that metabolites of imatinib may chelate with melanin and/or iron to cause pigment deposition in a manner similar to hydroxychloroquine, although this has not been specifically demonstrated for imatinib.^10^ In general, the pathogenesis behind hyperpigmentation remains poorly understood with multiple potential contributing clinical and pathologic patterns existing.

Treatment is challenging and requires risk-benefit discussion with the patient to balance therapy for malignancy with benign pigmentary changes of the skin. Hyperpigmentation of the nails and teeth has been reported to resolve after cessation of imatinib and similar improvements have been seen in cutaneous involvement.^4^^,^^5^ Kok and Chua^11^ demonstrated a successful case of treatment of imatinib-induced hyperpigmentation after 5 sessions of treatment with a picosecond alexandrite laser. In summary, imatinib is a rare but potential culprit for medication-induced hyperpigmentation.

## Conflicts of interest

None disclosed.
